# Supplementary material for: Impact of data quality on deep learning prediction of spatial transcriptomics from histology images
Source: Genome Biol. 2026 Aug 11;27:348. doi: 10.1186/s13059-026-04236-2 (PMC13746554; doi:10.1186/s13059-026-04236-2)
Supplement: Supplementary file 1 [file 13059_2026_4236_MOESM1_ESM.pdf]

Supplementary Figures

Impact of data quality on deep learning prediction of spatial transcriptomics from histology images

Caleb Hallinan[1,2], Calixto-Hope G. Lucas[3,4], Jean Fan[1,2,*]

[1]Center for Computational Biology, Whiting School of Engineering, Johns Hopkins University, Baltimore, MD 21211, USA

[2]Department of Biomedical Engineering, Johns Hopkins University, Baltimore, MD 21211, USA

[3]Department of Pathology, Johns Hopkins University, Baltimore, MD 21287, USA

[4]Department of Neurosurgery, Johns Hopkins University, Baltimore, MD 21287, USA

[*]Corresponding author: jeanfan@jhu.edu

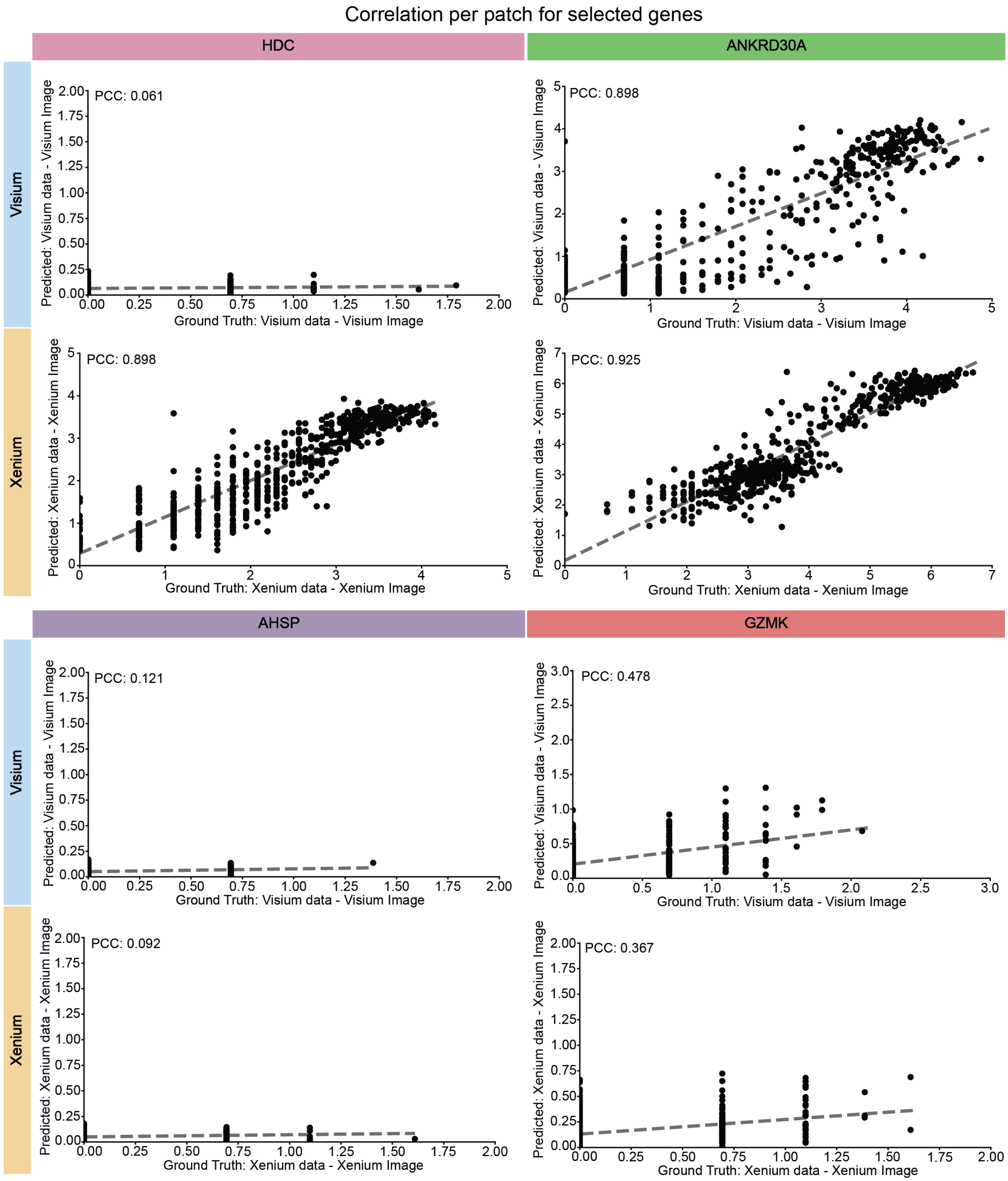

Fig. S1: Correlation between ground-truth and predicted gene expression across patches for selected genes. Scatterplots comparing the ground truth held-out test set patches with the predicted expression of patches for *HDC*, *ANKRD30A*, *AHSP*, and *GZMK*. The gray dotted line denotes the linear regression fit. Analyses shown are based on a single seed rather than five independently trained models.

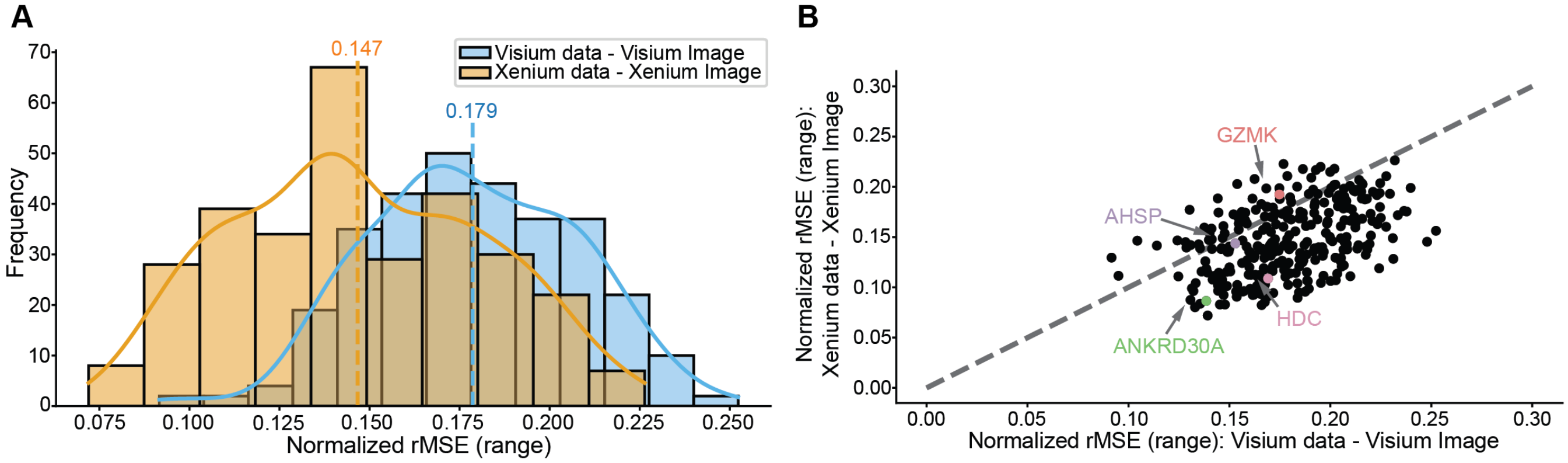

Fig. S2: Prediction performance using normalized rMSE for Visium vs. Xenium data. A. Histogram showing the distribution of normalized rMSE for gene expression predictions using Visium and Xenium data. The dotted vertical line denotes the mean rMSE, and the solid curved line traces the density estimate. Results are computed on the test set and represent the average performance across five independently trained models. **B.** Scatterplot comparing the normalized rMSE of predictions from Visium and Xenium data, based on the test set and averaged over five models. The gray dotted line denotes x=y.

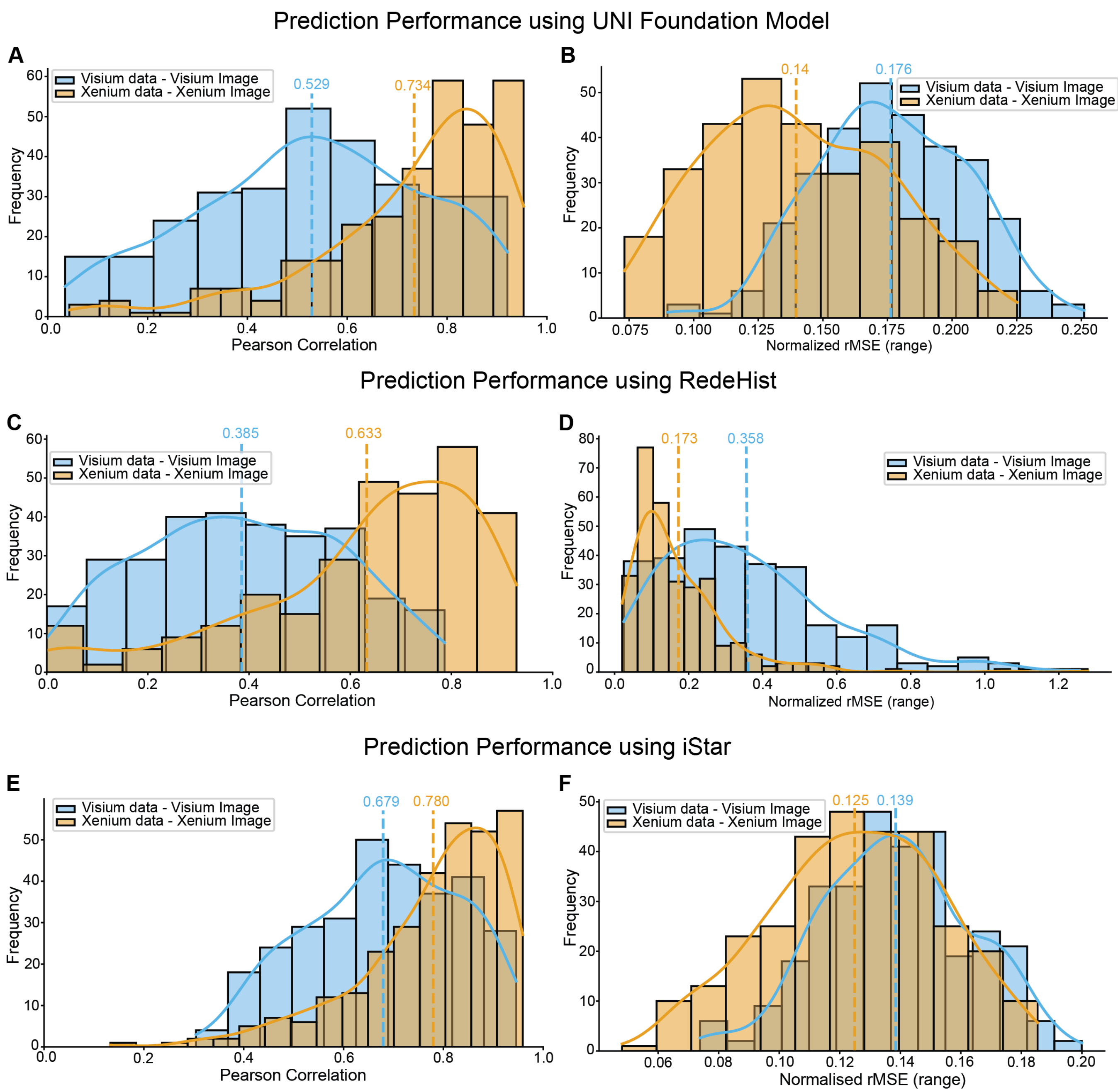

Fig. S3: Prediction performance across alternative model architectures and feature extractors. A-B. Distribution of Pearson correlation (A) and normalized rMSE (range) (B) for gene expression predictions trained on Visium and Xenium data using the UNI foundation model. **C-D.** Same as A-B using the RedeHist model. **E-F.** Same as A-B using the iStar model. In all panels, the dotted vertical line denotes the mean and the solid curve traces the density estimate. Results for UNI represent the average across five independently trained models evaluated on the test set; results for RedeHist and iStar are computed on the full dataset.

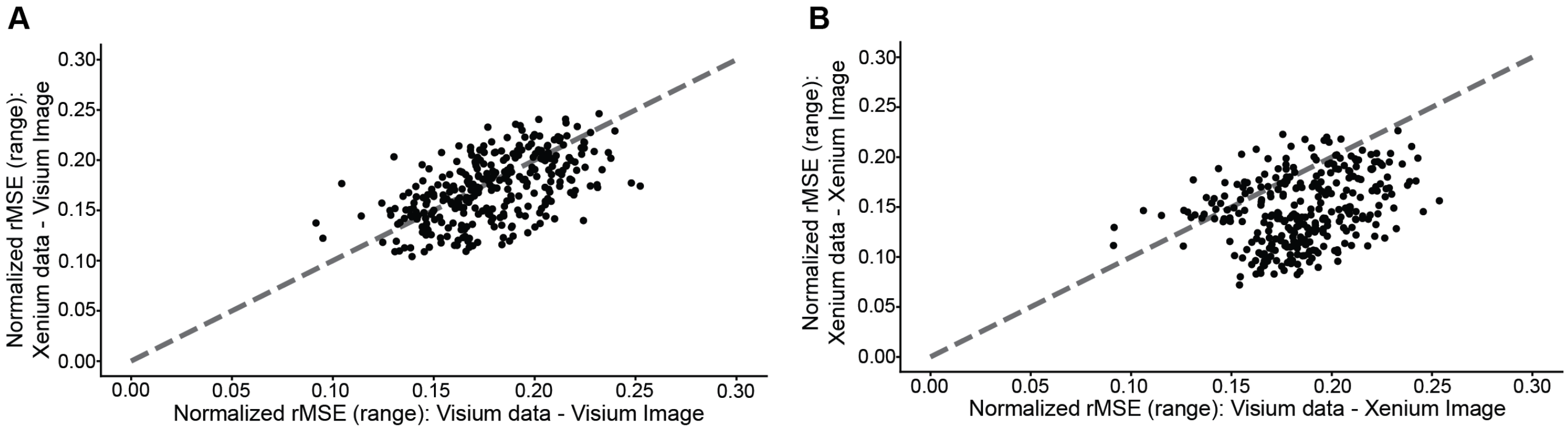

Fig. S4: Prediction performance using normalized rMSE for Visium vs. Xenium molecular data. Scatterplots of normalized rMSE for models trained on varied molecular inputs, evaluated on the held-out test set and averaged across five independent runs, using (A) the Visium histology image and (B) the Xenium histology image. The gray dotted line denotes x=y.

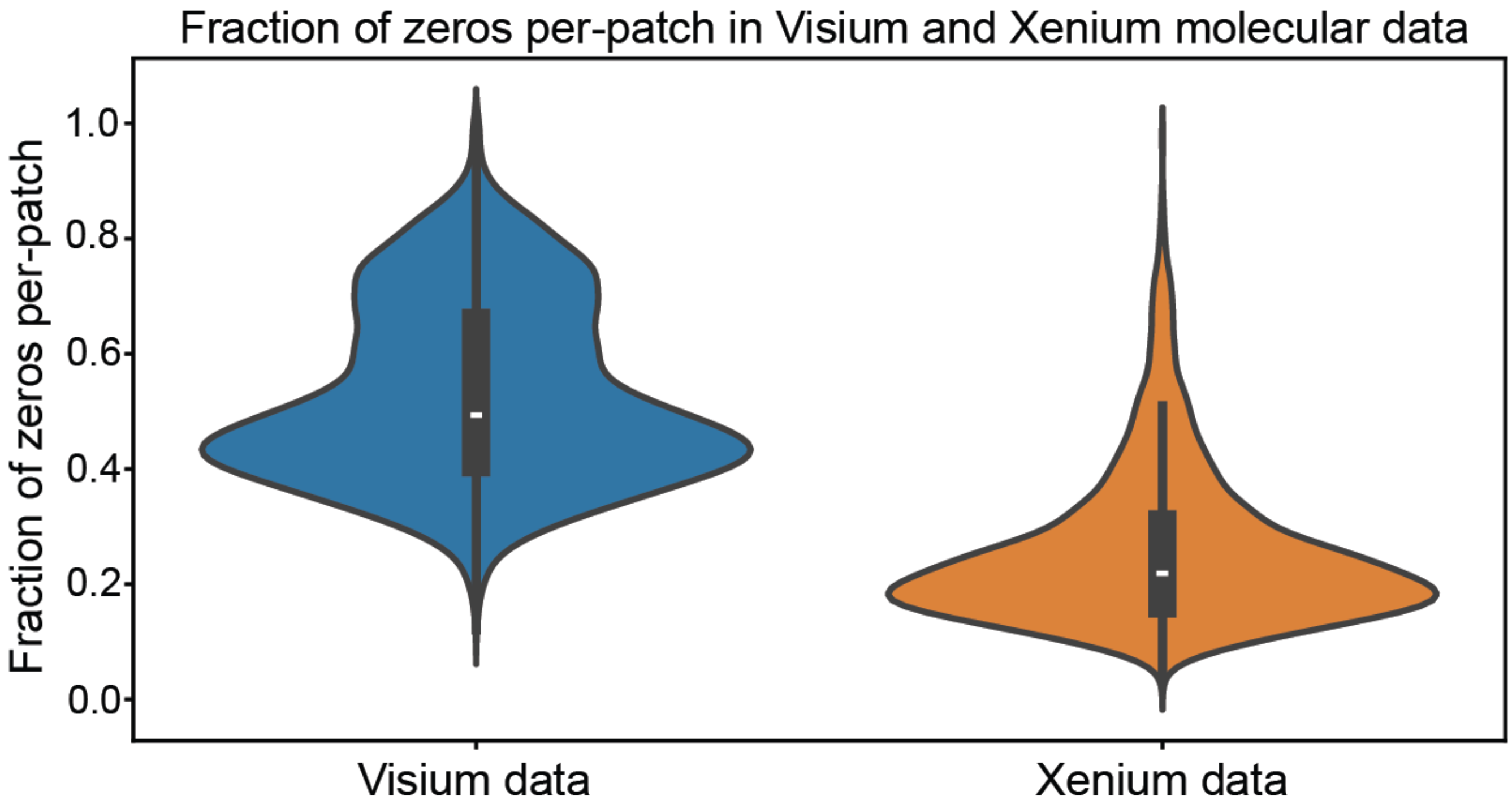

Fig. S5: Sparsity in Visium and Xenium molecular data. Violin plots of the per-patch fraction of zero counts in Visium and Xenium molecular data. The shape of each violin reflects the density of values along the y-axis, and the overlaid boxplot indicates the median and the 25th and 75th percentiles.

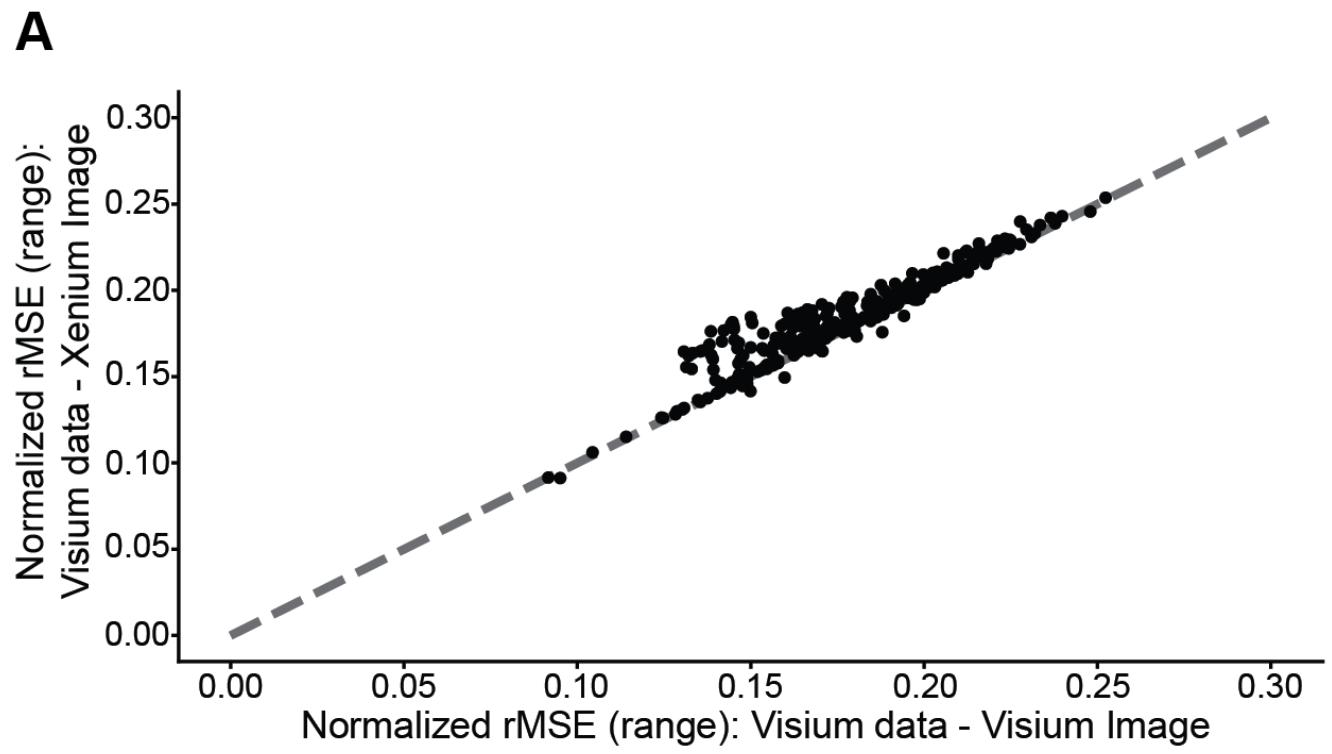

Fig. S6: Prediction performance using normalized rMSE for Visium vs. Xenium imaging data. Scatterplots of normalized RMSE for models trained on varied image inputs, evaluated on the held-out test set and averaged across five independent runs, using (A) the Visium molecular data and (B) the Xenium molecular data. The gray dotted line denotes x=y.

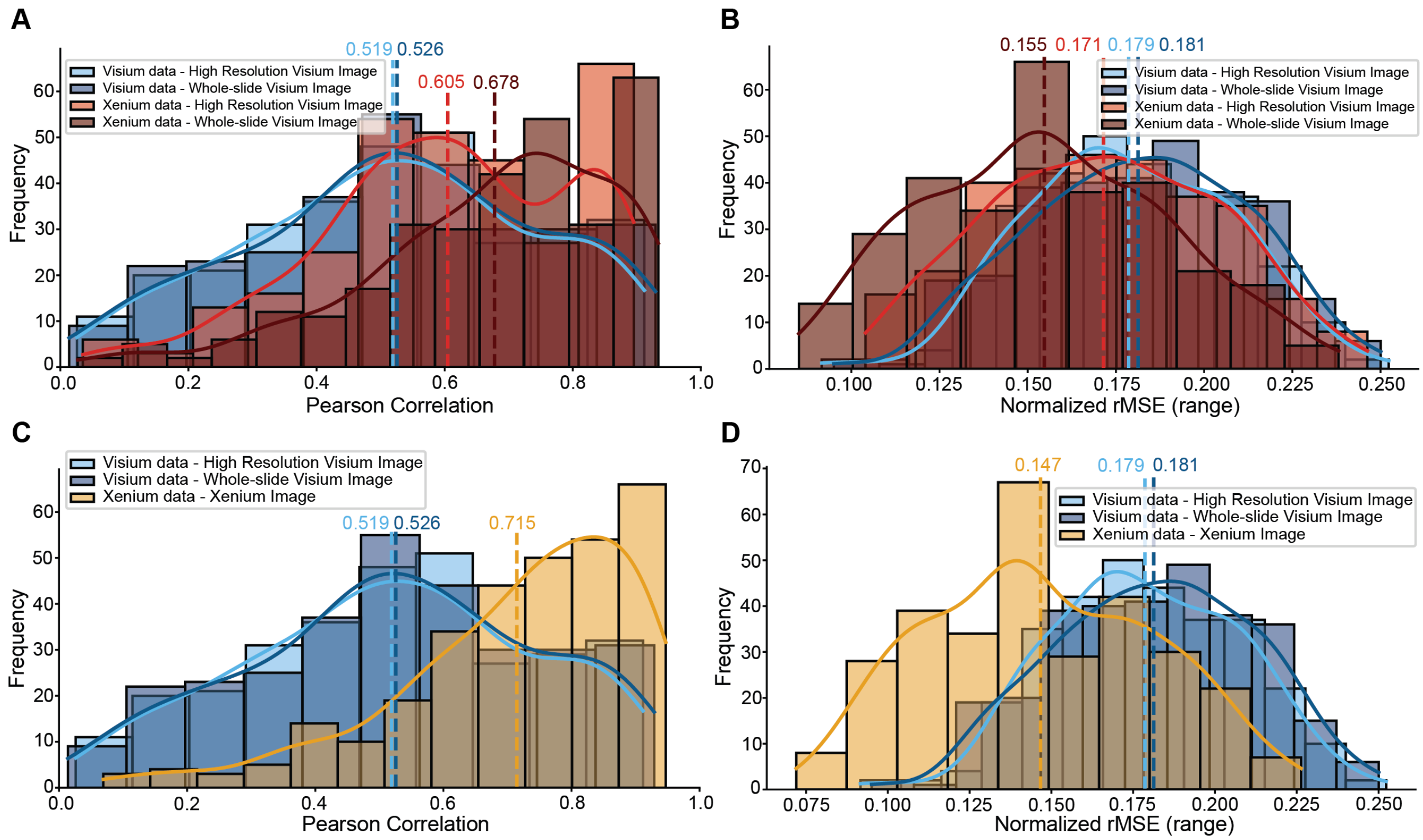

Fig. S7: Prediction performance using the whole-slide Visium image. A-B. Distribution of Pearson correlation (A) and normalized rMSE (B) for gene expression predictions using Visium and Xenium molecular data, each paired with either the high-resolution Visium image or the Visium whole-slide image. **C-D.** Distribution of Pearson correlation (C) and normalized rMSE (D) for gene expression predictions using Visium molecular data with both Visium images, alongside Xenium molecular data with the Xenium image. In all panels, the dotted vertical line denotes the mean and the solid curve traces the density estimate. Results represent the average across five independently trained models evaluated on the test set.

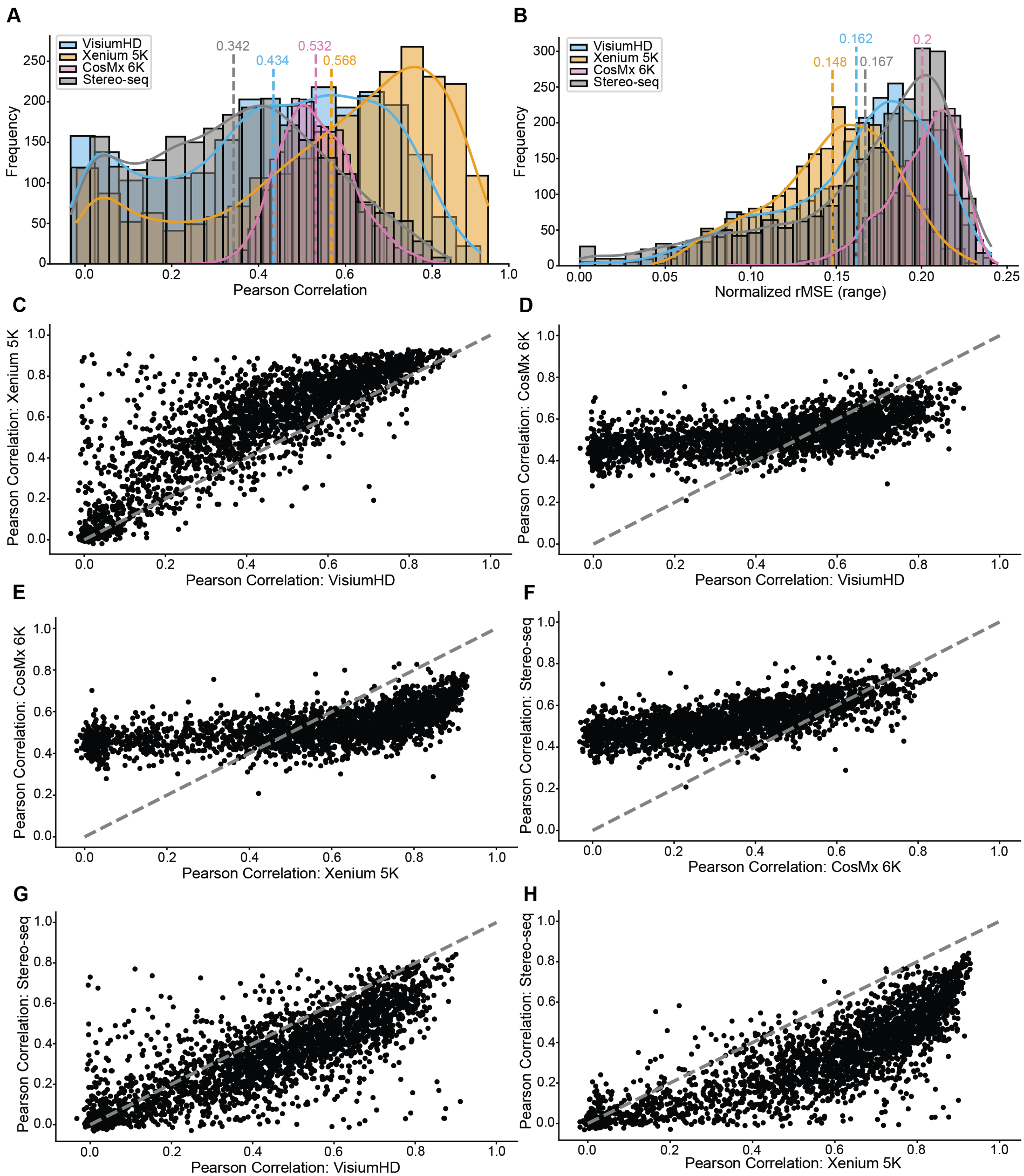

Fig. S8: Prediction performance on the colon adenocarcinoma dataset. A-B. Distribution of Pearson correlation (A) and normalized rMSE (B) for gene expression predictions trained on VisiumHD, Xenium 5K, CosMx 6K, and Stereo-seq data. The dotted vertical line denotes the mean and the solid curve traces the density estimate. **C-H.** Scatterplots comparing per-gene Pearson correlation between technology pairs: VisiumHD vs. Xenium 5K (C), VisiumHD vs. CosMx 6K (D), Xenium 5K vs. CosMx 6K (E), CosMx 6K vs. Stereo-seq (F), VisiumHD vs. Stereo-seq (G), and Xenium 5K vs. Stereo-seq (H). The gray dotted line denotes x=y. All results represent the average across five independently trained models evaluated on the test set.

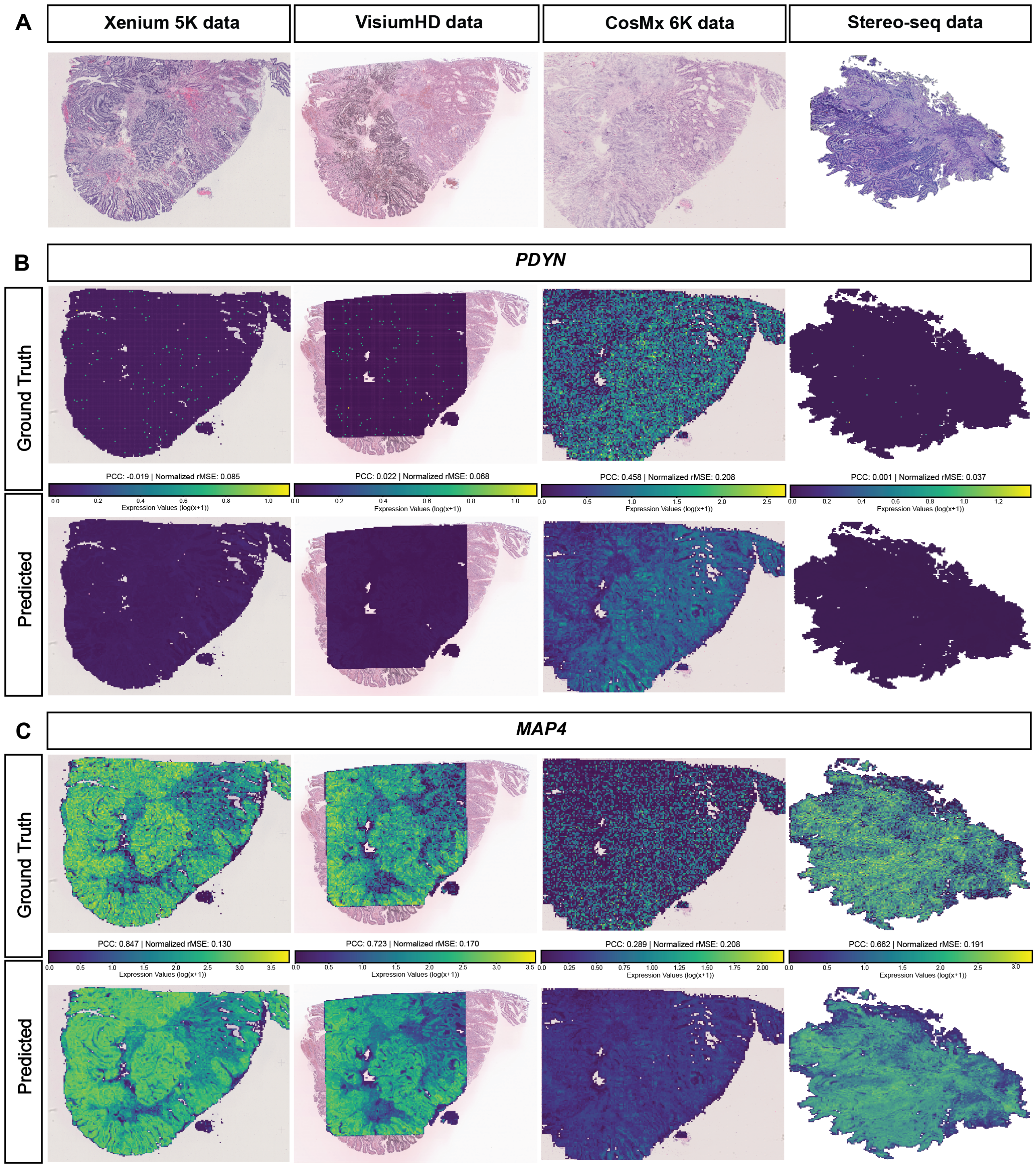

Fig. S9: Examples of gene prediction performance on the colon adenocarcinoma dataset. A. Whole-slide H&E images associated with the Xenium 5K, VisiumHD, CosMx 6K, and Stereo-seq spatial transcriptomics technologies. **B.** Ground-truth and predicted spatial expression of the *PDYN* gene across Xenium 5K, VisiumHD, CosMx 6K, and Stereo-seq datasets. **C.** Ground-truth and predicted spatial expression of the *MAP4* gene across Xenium 5K, VisiumHD, CosMx 6K, and Stereo-seq datasets.

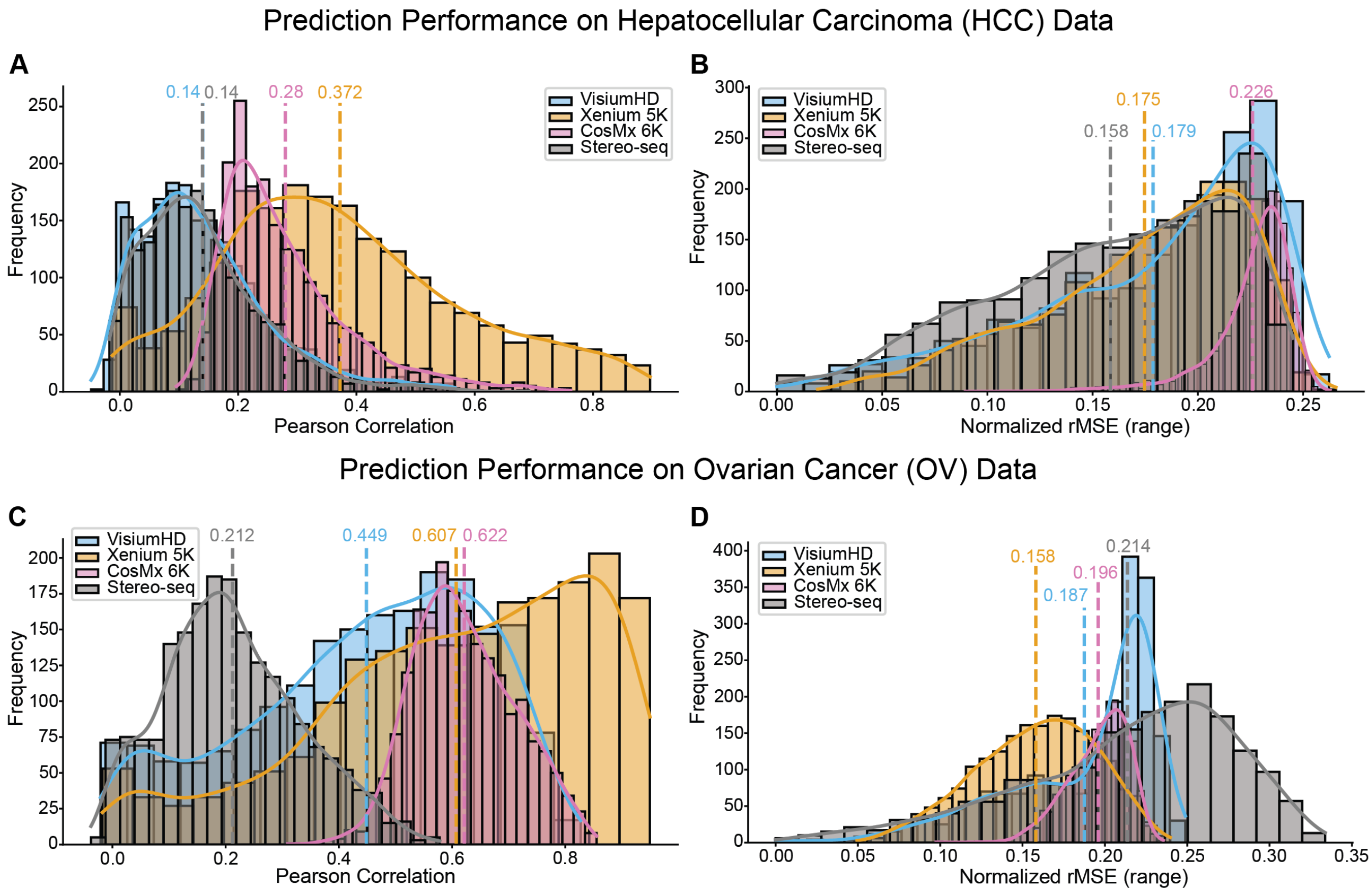

Fig. S10: Prediction performance the hepatocellular carcinoma and ovarian cancer datasets. A-B. Distribution of Pearson correlation (A) and normalized rMSE (B) for gene expression predictions trained on VisiumHD, Xenium 5K, CosMx 6K, and Stereo-seq data from hepatocellular carcinoma (HCC) tissue. **C-D.** Same as A-B for ovarian cancer (OV) tissue. In all panels, the dotted vertical line denotes the mean and the solid curve traces the density estimate. Results represent the average across five independently trained models evaluated on the test set.

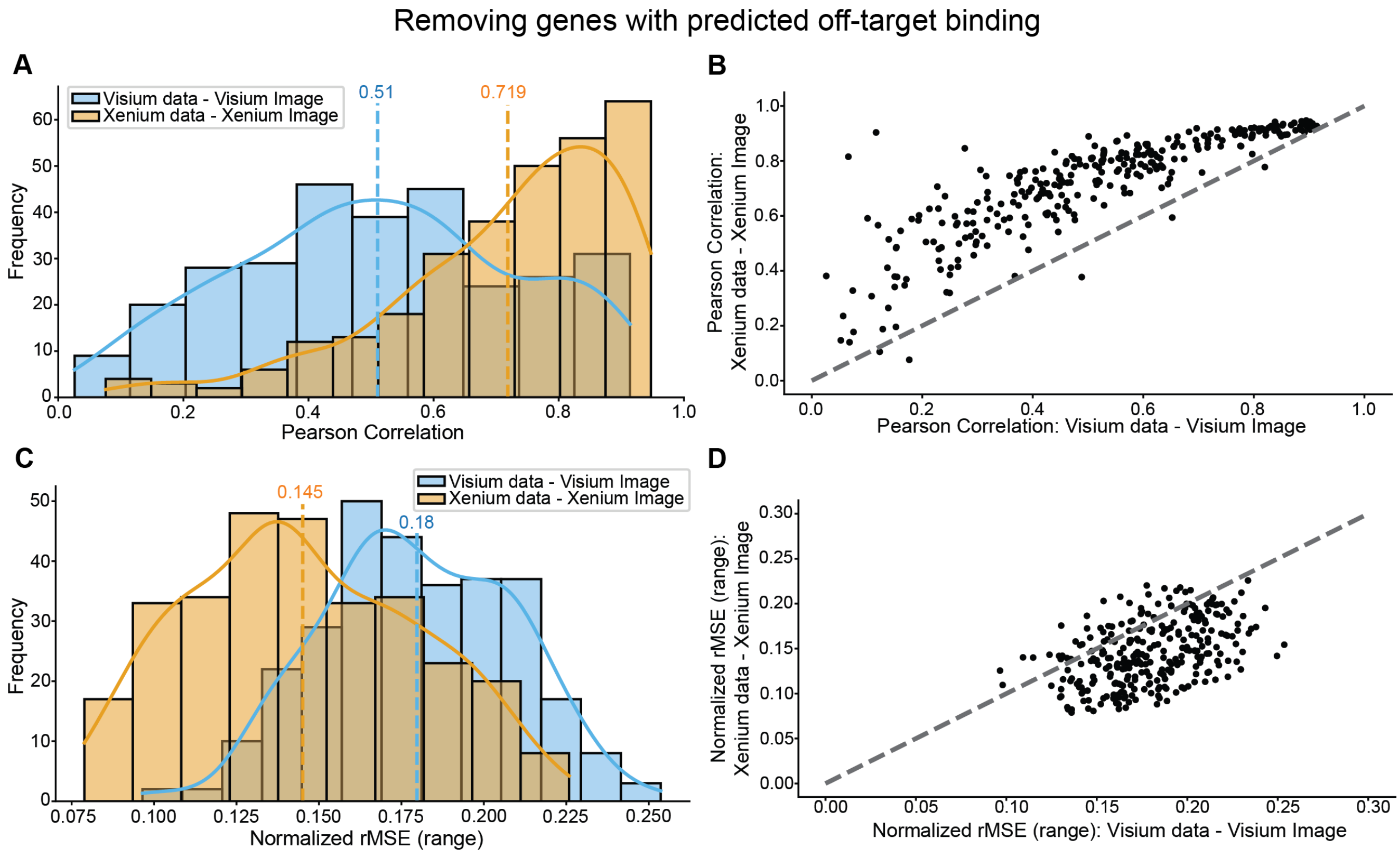

Fig. S11: Visium vs. Xenium results when removing genes with predicted off-target binding. A. Histogram showing the distribution of Pearson correlation for gene expression predictions using Visium and Xenium data. The dotted vertical line denotes the mean Pearson Correlation, and the solid curved line traces the density estimate. Results are computed on the test set and represent the average performance across five independently trained models. **B.** Scatterplot comparing the Pearson correlation of predictions from Visium and Xenium data, based on the test set and averaged over five models. The gray dotted line denotes x=y. **C.** Histogram showing the distribution of normalized rMSE for gene expression predictions using Visium and Xenium data. The dotted vertical line denotes the mean rMSE, and the solid curved line traces the density estimate. Results are computed on the test set and represent the average performance across five independently trained models. **D.** Scatterplot comparing the normalized rMSE of predictions from Visium and Xenium data, based on the test set and averaged over five models. The gray dotted line denotes x=y.

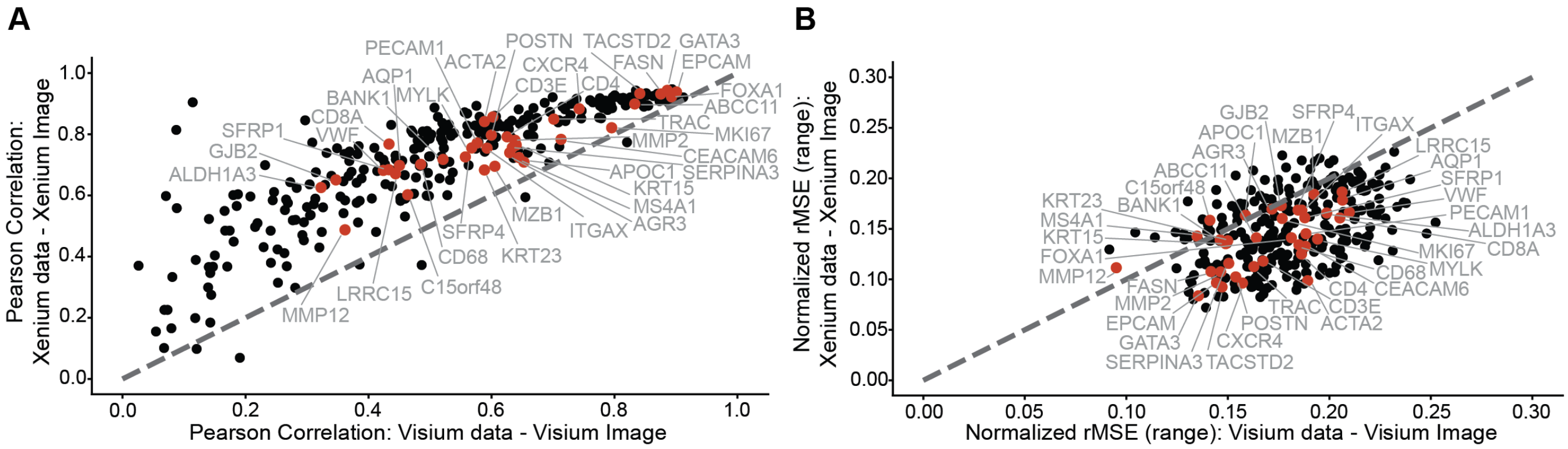

Fig. S12: Cell-type marker gene prediction performance. A. Scatterplot comparing the Pearson correlation of predictions from Visium and Xenium data, based on the test set and averaged over five models. The gray dotted line denotes x=y, and cell-type marker genes are highlighted in red and labeled. **B.** Scatterplot comparing the normalized rMSE of predictions from Visium and Xenium data, based on the test set and averaged over five models. The gray dotted line denotes x=y, and cell-type marker genes are highlighted in red and labeled.

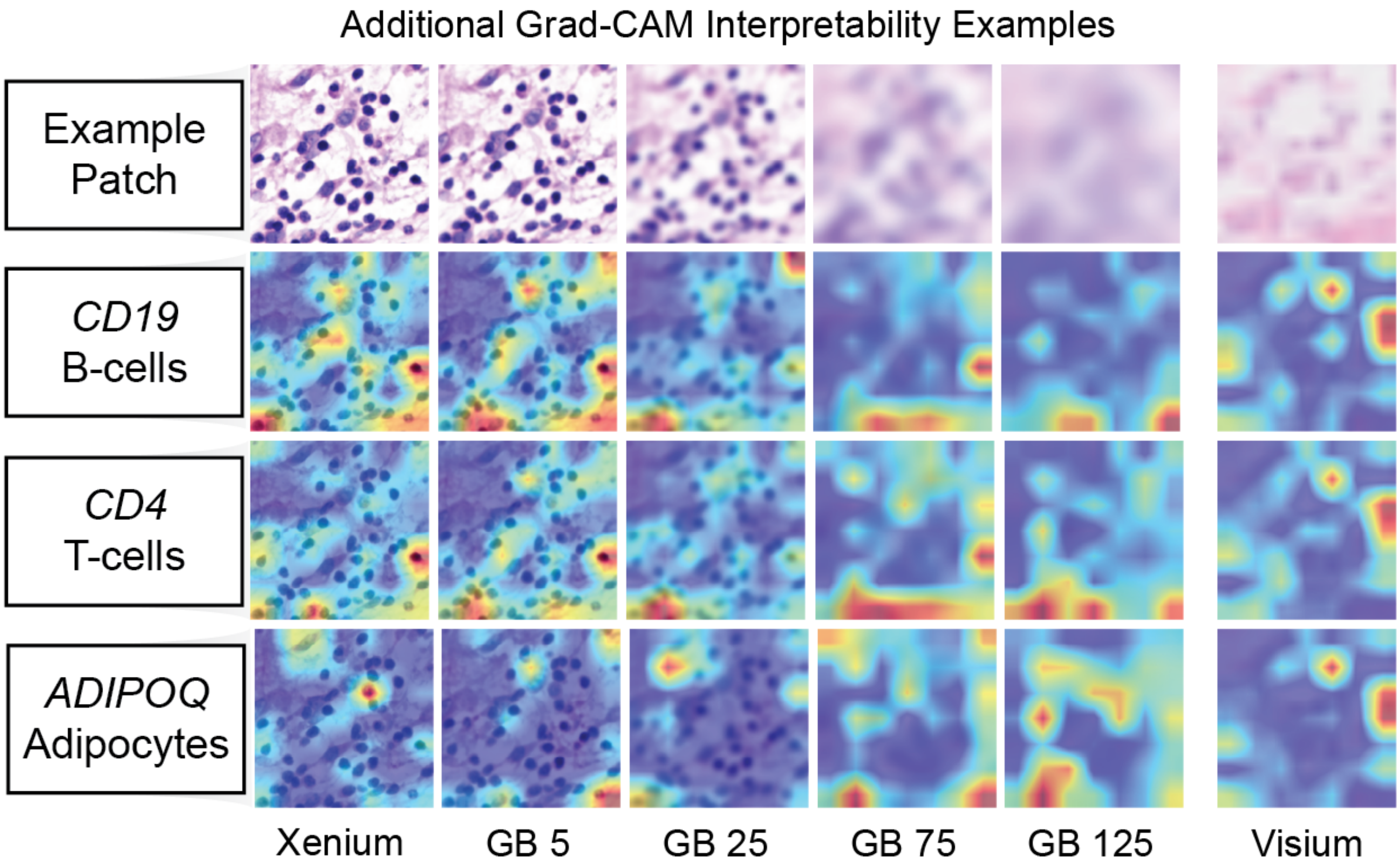

Fig. S13: Additional Grad-CAM interpretability examples. A representative histology patch is shown under increasing Gaussian blur (GB) levels, with corresponding Grad-CAM heatmaps for *CD19* (B-cell marker), *CD4* (T-cell marker), and *ADIPOQ* (adipocyte marker).
